# A multimodal tableware system for supporting mindful eating in solo dining: a pilot study

**DOI:** 10.3389/fpubh.2026.1830317

**Published:** 2026-08-17

**Authors:** Ming Zhang, Shan Ding, Zhihui Zhang, Qinglong Kong, Xun Li

**Affiliations:** School of Design and Arts, Beijing Technology and Business University, Beijing, China

**Keywords:** digital health intervention, eating behavior regulation, mindful eating, multimodal feedback, solo dining

## Abstract

**Background:**

Promoting healthier eating behaviors is an important strategy for addressing the global burden of lifestyle-associated diseases. With the increasing prevalence of solo dining, individuals often develop rapid and inattentive eating habits, which may contribute to poor digestion, overeating, and unhealthy dietary patterns.

**Objective:**

To explore how improving solo dining behaviors can be embedded in everyday dining artifacts, we developed SensEat, a modular embodied tableware system that coordinates visual, auditory, and haptic micro-feedback during solo dining.

**Materials and methods:**

An exploratory fixed-sequence within-subject study involving 20 participants was performed to compare the SensEat-guided intervention (SGI) with an app-guided intervention (AGI). Because all participants completed AGI prior to SGI, the condition and order effects could not be separated. Behavioral outcomes (meal duration and chews per bite), self-reported experience (five IMI-informed single-item ratings), and perceived task workload (three selected NASA-TLX ratings) were assessed.

**Results:**

Relative to the AGI session, the SGI session was associated with longer meal duration and more chews per bite (both P_Holm_ < 0.001), higher Interest/Enjoyment (P_Holm_ = 0.016) and Value/Usefulness (P_Holm_ = 0.002), and higher Mental Demand (P_Holm_ = 0.013). Differences in Perceived Competence, Effort/Importance, Pressure/Tension, Performance, and Frustration were not statistically significant after Holm correction.

**Conclusion:**

These exploratory findings support the feasibility of embedding multimodal eating-pace guidance into everyday tableware. Within the fixed AGI → SGI sequence, the SensEat session is associated with behavioral patterns such as slower eating, higher Interest/Enjoyment and Value/Usefulness, and higher Mental Demand, which highlights the promise and attentional cost of artifact-embedded guidance.

## 1 Introduction

Promoting sustainable health behaviors, particularly lifestyle hygiene practices, has become an important priority in addressing global challenges related to lifestyle-associated diseases (1). Eating behaviors represent routine lifestyle activities with cumulative long-term health consequences (2), highlighting the need for interventions that can be naturally embedded into everyday contexts (3). During the past decade eating behaviors have significantly changed because of the fast-paced and multitasking social environment. The increase in single-person households and transformations in social structure have caused the prevalence of the “solo dining” phenomenon, which makes individualized and contextualized eating patterns a new norm (4). Individuals who engage in solo dining exhibit distinct characteristics in their eating motivation, pace, and food choices, indicating shifts in dietary psychology and health management in modern society (5). Meanwhile, the widespread use of mobile devices has linked eating behaviors to screen-related activities, such as browsing information, working, and engaging in social interactions while eating, which may induce “mindless eating.” (6) Such behavior reduces attention to food intake and sensory feedback, thereby increasing the risks of overeating, digestive disorders, and obesity (7). Long-term unhealthy eating habits have been identified as a major risk factor of non-communicable diseases (NCDs) (8). The United Nations Sustainable Development Goals (SDGs), particularly SDG 3 (Good Health and Wellbeing), emphasize the importance of fostering healthy daily practices and preventing non-communicable diseases through accessible and contextually integrated interventions (9). Solo dining is not only a social phenomenon but also closely associated with individuals’ psychological states, attentional allocation, and health-related behaviors (10). In typical solo-dining environments, such as university cafeterias, office spaces, and dormitories, environmental stimuli and psychological factors further disrupt eating rhythms, making healthy dietary regulation more challenging (11). Solo dining may lead to mindless eating and insufficient dietary self-regulation (6).

Emerging research focuses on how to promote mindful and healthy eating behaviors in daily contexts. A commonly adopted strategy involves the use of self-monitoring systems and digital feedback to help individuals enhance awareness and behavioral control during meals (12). For instance, mobile applications that provide real-time feedback on eating rates have been shown to effectively improve eating pace and intake, thereby enhancing satiety and reducing energy consumption (13). Similarly, longer chewing and slower eating have demonstrated positive effects on digestive function and psychological satisfaction based on the studies employing visual or auditory timing cues (14, 15). Various technologies have been explored to promote mindful eating, such as mobile health–based dietary regulation programs (16), dining environment designs incorporating environmental or social cues (17), and smart utensil systems capable of sensing and regulating eating behaviors (18). However, limited adaptability, insufficient immersion, and considerable usability burdens restrict these interventions’ effectiveness and scalability in real-world solo dining contexts. In the absence of social interaction, individuals’ attention tends to be directed more toward the food itself or other external stimuli, such as mobile phones or television, which alters the emotional and perceptual processes of the eating experience (19). Psychologically, solo diners often exhibit two contrasting attentional patterns. Some individuals experience loneliness due to social isolation, leading to attentional distraction and potentially mindless eating (20), whereas others focus more intently on the taste and texture of food, thereby developing heightened sensory awareness (21). Such differences may be influenced by individual personality traits, cultural background, and dining purposes. In the context of technology use, Lemke and Schifferstein suggested that information and communication technology (ICT) devices acted as “virtual companions” during solo dining (6). Although electronic devices can alleviate loneliness, these devices may divert diners’ attention away from food to reduce eating mindfulness and satisfaction. Moreover, Choi et al. found that frequent solo diners displayed greater affective variability and tended to compensate for the absence of social interaction through digital media and other sensory stimuli (22). Solo dining behaviors reveal a complex and dynamic relationship between attention and emotional experience. The attention of solo diners may be either highly focused on sensory experience or easily distracted by external stimuli, which provides a novel perspective for research in human-computer interaction design and digital health interventions. Therefore, investigating multimodal and embodied interaction approaches in dining environment provides both scientific and practical value for sustainable health promotion.

From a design perspective, embedding behavioral support into everyday artifacts offers a potential pathway for integrating health-related guidance into routine activities (23). Smart tableware and embodied interaction technologies have demonstrated remarkable innovative potential in the field of dietary behavior intervention. By integrating sensors, real-time data feedback, and interactive interfaces, smart tableware provides users with personalized dietary recommendations and behavioral guidance. For example, interactive utensils equipped with weight sensors can automatically detect food intake through mobile applications or gamification mechanisms to encourage children to concentrate on the eating process, reduce picky eating behaviors, and improve the diversity and balance of nutritional intake (24). Empirical studies have shown that interactive tableware systems not only enhance children’s eating focus but also effectively improve their dietary habits in the short term. (25) Among adults and individuals with chronic diseases, smart tableware is often designed as portioned plates or calibrated utensils, which use visual cues and capacity constraints to guide users in balancing the intake proportions of carbohydrates, proteins, and vegetables. It suggests that the use of such tableware can significantly reduce energy intake, decrease body weight, and improve metabolic indicators such as blood glucose levels (26). However, some studies have also implied that merely relying on tableware size adjustment may lead to compensatory eating behaviors, and the long-term effectiveness of such interventions requires further validation (27). Embodied interaction enhances the proactivity and self-awareness of behavior change by closely integrating behavior monitoring and real-time feedback with users’ physical movements (28). Digital interventions incorporating behavior change techniques such as goal setting, social support, and prompts or cues have demonstrated improved engagement and adherence, particularly among adolescents and young adults (29). By embedding healthy eating goals within interactive activities, these approaches can enhance engagement and long-term adherence to healthy behaviors (30). Compared with instruction-driven approaches, artifact-mediated interventions embedded within everyday environments may improve ecological validity and user acceptance in real-world contexts, thereby supporting the feasibility of sustained lifestyle practices (31).

Mindful eating may be considered a form of lifestyle hygiene practice, as it involves routine self-regulation behaviors that support both physical and psychological wellbeing in daily life (32). In the context of solo dining, implicit and multimodal intervention strategies have recently aimed to promote the spontaneous transformation of healthy eating behaviors through noninvasive, environmentally embedded, and multisensory approaches. Implicit interventions emphasize subtly guiding individuals’ choices and behaviors through environmental cues, default options, food placement, and menu design, rather than explicit education or coercive measures. Implicit intervention techniques can effectively increase the selection and consumption of healthy food without raising diners’ awareness (33). Among these methods, food placement and default options have shown the most significant effects, whereas naming and labeling interventions have been relatively limited, suggesting the necessity of expanding the application of implicit interventions in diverse dining contexts (34). Furthermore, multimodal interventions enhance the effectiveness of behavioral interventions by integrating multiple sensory stimuli and behavioral regulation techniques. In solo dining contexts, information and communication technologies (ICTs) have been employed to enhance the dining experience and alleviate loneliness and also contribute to unhealthy food choices and reduced satiety (6). Recent studies suggest that the physicalization of sensor data combined with self-annotation can help individuals reflect on their eating behaviors, enhance mindful eating awareness, and facilitate self-regulation of healthy habits (35). For older adults living alone, multimodal interventions that integrate social interaction, nutritional guidance, and cooking skills training have been shown to significantly improve self-efficacy, dining enjoyment, and energy intake especially among women (36). Notably, the psychological health risks associated with solo dining also warrant attention. Studies have revealed that involuntary solo dining is closely associated with mental health issues such as depression, indicating that health interventions should incorporate social connection and psychological support to prevent the exacerbation of loneliness-induced negative eating behaviors (37).

In response to the growing demand for sustainable lifestyle interventions that can be seamlessly integrated into daily routines, this study focuses on the mechanisms of dietary behavior regulation in solo dining contexts, integrating interdisciplinary perspectives from Human–Food Interaction (HFI), intelligent tableware design, embodied perception, and implicit intervention. Digital technologies designed to promote mindful eating and regulate eating pace have been extensively explored in Human-Computer Interaction (HCI) and behavioral health research. Previous studies mainly relied on screen-based feedback systems to raise users’ awareness of eating behaviors and support self-monitoring, such as mobile apps that provide eating rate feedback and visual timers for bite pacing (38, 39). Although these feedback systems demonstrated positive effects on increasing eating awareness and reducing food intake, their reliance on screen-based prompts often distracted users from the dining experience (38). Thus, recent HCI research has shifted to embodied and ambient interventions, where feedback is seamlessly integrated into the dining context, including the tableware that measures and subtly guides chews per bite and the utensils that provide vibrotactile cues for mindful eating (40). It suggests that embedding multimodal cues into physical artifacts can encourage healthier eating habits with lower cognitive load (17, 41). However, more studies are needed to compare multimodal and embodied interventions with traditional screen-based feedback particularly in the context of solo dining, where social absence and screen distraction are prevalent (42, 43).

Previous studies have examined the behavioral outcomes of smart utensils, however it is still unclear how multimodal interactions affect users’ experiential dimensions, such as enjoyment, perceived companionship, and emotional engagement during solitary meals (44, 45). To address this gap, we propose SensEat, a modular smart tableware system that integrates embedded sensing units with multimodal and micro-intervention feedback mechanisms. This system is designed to subtly and contextually regulate users’ eating behaviors by providing visual, auditory, and haptic cues that encourage slower eating pace and increased chews per bite. SensEat aims to naturally support healthy eating habits within daily dining interactions based on the design principles of micro-intervention, low intrusiveness, and social friendliness. To explore the feasibility, user experience, and session-level behavioral differences associated with SensEat, we deployed SensEat in real-world solo dining environments with 20 participants and conducted a mixed-method study including behavioral tracking, questionnaire assessments, and in-depth interviews. Within this exploratory fixed-sequence comparison, we examined the behavioral, experiential, and practical implications of embedding multimodal feedback into everyday tableware. This work contributes to three aspects in Human–Food Interaction research. First, it presents a modular, sensor-integrated tableware prototype that coordinates ambient light, auditory, and haptic micro-feedback to deliver eating-pace guidance without requiring continuous screen attention. Second, it provides an exploratory, system-level active-comparator account of behavioral, self-reported experiential, and workload differences between SensEat and an app-guided system in solo-dining settings. Third, it derives design knowledge concerning the opportunities and tensions of embodied multimodal guidance, including perceived companionship and enjoyment, variability in sensory preferences, and the trade-off between experiential value and mental demand. Our study was guided by the following research questions:

*RQ1*: In what ways do eating pace and related behaviors differ between the AGI and SGI sessions?

*RQ2*: How do participants’ solo-dining experiences differ between the two sessions, and how do they describe the distinctive experiential qualities of SensEat?

*RQ3*: How does perceived workload differ between the two sessions, and what practical challenges do participants report when using SensEat?

## 2 Materials and methods

### 2.1 The design of SensEat

**Figure 1:**
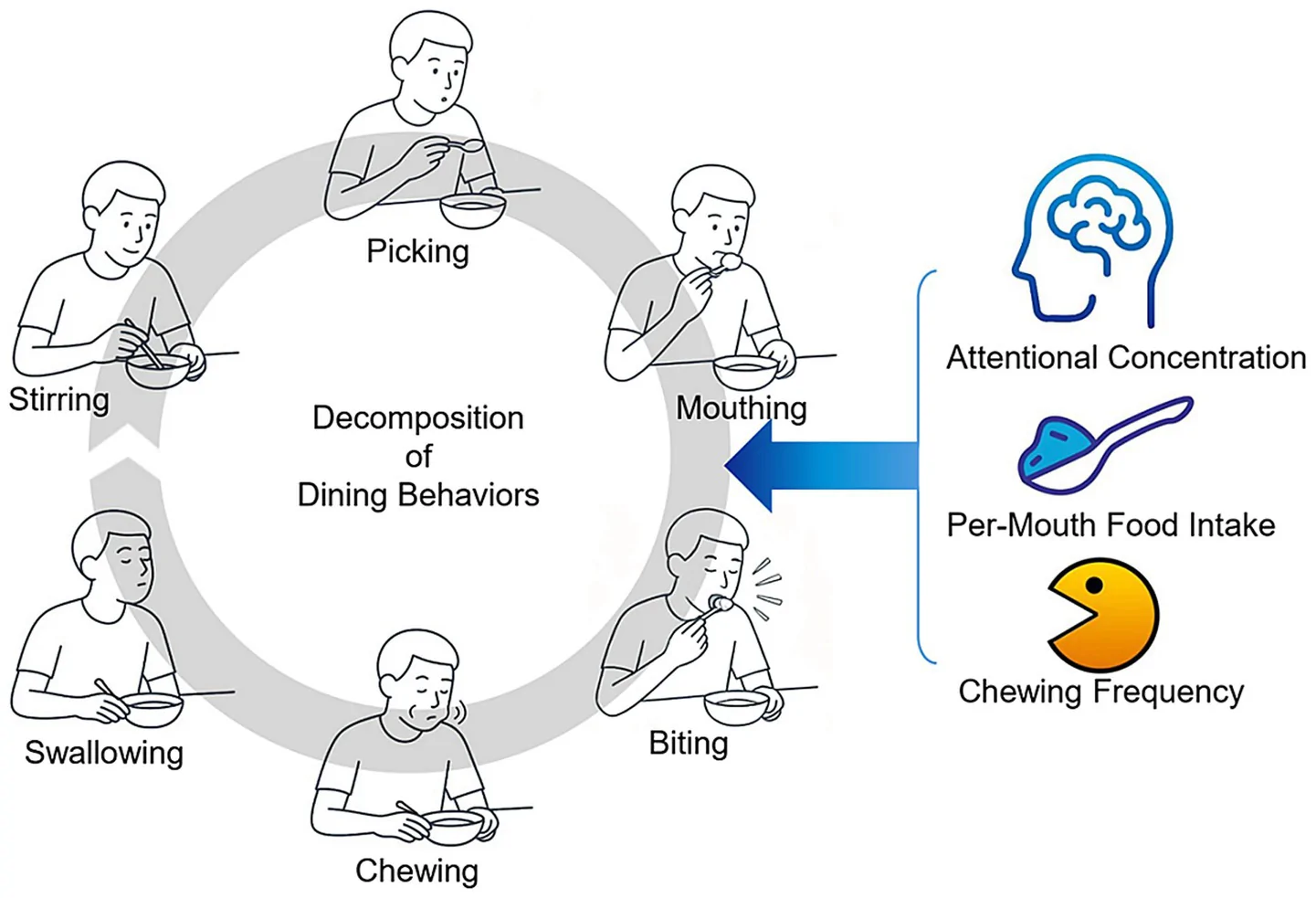
Decomposition of dining behaviors and the key influencing factors.

Based on the random sampling of 10 complete eating videos from participants, we conducted qualitative annotation and decomposed the process into a typical action sequence of “stirring - picking up - mouthing - biting - chewing - swallowing” (Figure 1). Through this analysis, attention concentration, per-mouth food intake, and chews per bite were identified as key factors influencing eating behavior (46–48). Building upon these findings, the system was designed to establish an implicit feedback mechanism intended to support users’ regulation of eating pace without interrupting their natural dining flow. Fogg Behavior Model (FBM) from behavioral science was adopted as the theoretical foundation for the design of the intelligent tableware system. The FBM posits that human behavior occurs through the joint interaction of three elements: motivation, ability, and trigger. The system concept was interactively designed according to these three components, and its feasibility and perceived acceptability were explored in real-world dining contexts. SensEat is designed to capture users’ daily eating rhythms and to trigger multimodal micro-feedback cues based on real-time data, thereby assisting them in recognizing and adjusting their eating behaviors.

**Figure 2:**
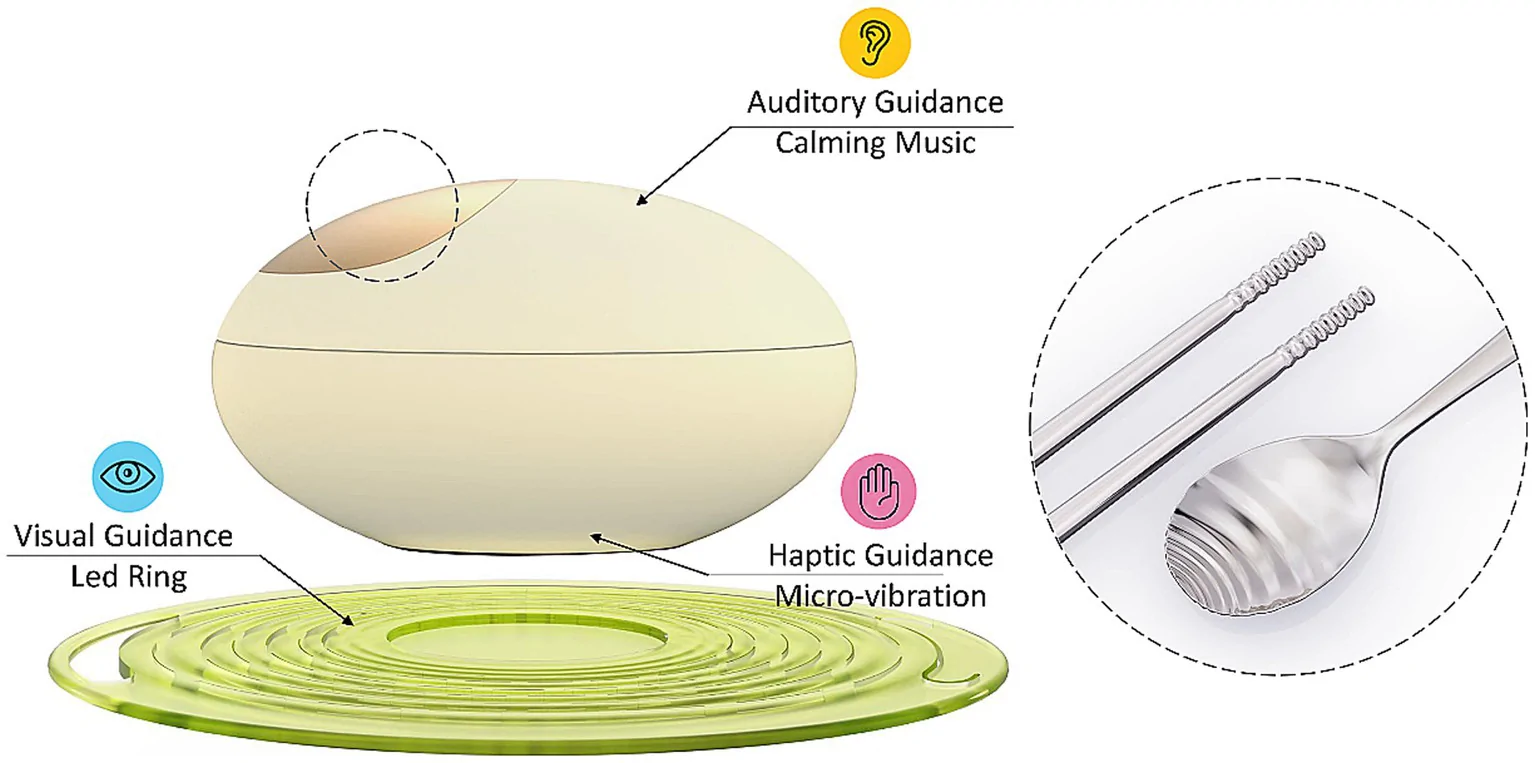
The design concept of SensEat.

In terms of motivation enhancement, individuals dining alone are often influenced by emotional states and external environmental factors, making it difficult to maintain sustained attention (19). To address this issue, the concept of Mindful Eating was introduced to enhance the dining experience. As a beneficial behavioral health intervention, Mindful Eating has been empirically shown to significantly increase dining satisfaction and reduce mindless eating (49). As shown in Figure 2, the semantic meaning of product form was leveraged in the design: the lunch box was shaped with a “grain” motif, symbolizing the origin and gentle rhythm of eating, while the placemat surface adopted a “ripple” texture to evoke a tranquil and focused dining atmosphere. Meanwhile, the spoon and chopstick tips were designed with a rippled texture to enhance oral tactile perception and facilitate behavioral feedback in the dimension of sensory awareness. Through these product-form features, the design was intended to support users’ motivation to engage with eating-pace guidance.

**Figure 3:**
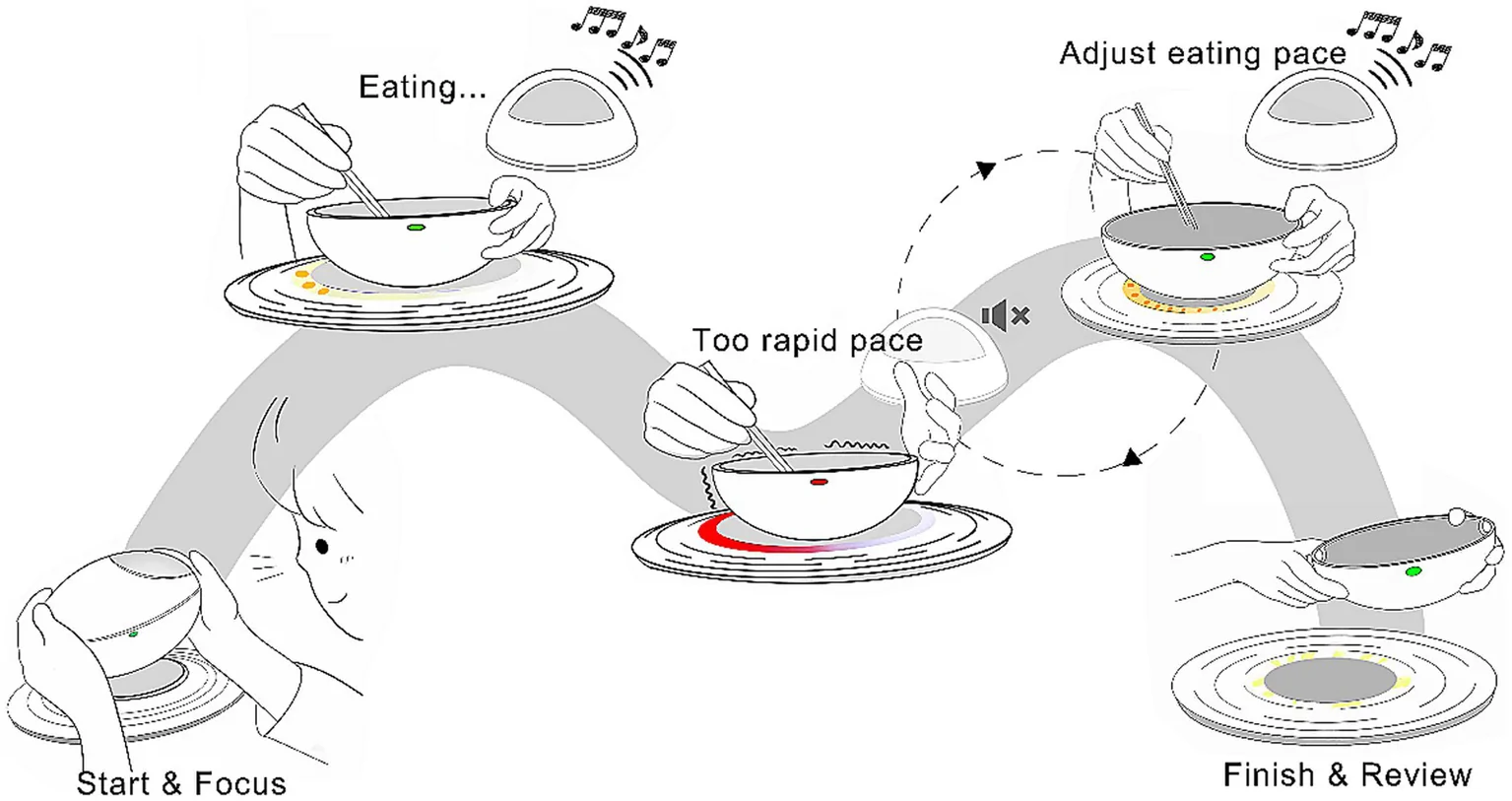
The interaction logic of the SensEat.

The interaction logic of the SensEat design is as illustrated in Figure 3. To lower the ability threshold, the entire dining process was decomposed into a series of achievable micro-tasks. For the present prototype, a 20-min meal-duration target was selected as an operational design parameter. Following task-decomposition principles (50), this target was divided into twenty 1-min micro-tasks. When the user began eating, one LED bead on the placemat automatically lit up every 60 s, providing a clear visual cue of progress in rhythmic eating. Accordingly, the minute-by-minute LED progression was implemented as a simple visual pacing scaffold, consistent with evidence that visual feedback can influence eating rate (51).

For the trigger design, a multimodal interaction approach (Table 1) was adopted, embedding behavioral triggers within the physical interaction interface. When the weight sensor detects food consumption, the system initiates the playback of soothing background music to establish an auditory cue for rhythm guidance. Previous objective measurements of eating rate have reported rates of approximately 50 g/min among individuals classified as fast eaters, compared with approximately 30–35 g/min among slower eaters (52). Based on this published reference, the prototype used an intake rate of >50 g/min (0.83 g/s) as a pragmatic operational threshold for activating real-time feedback. This mass-based threshold could be implemented directly using the integrated weight sensor and was applied consistently because all participants consumed the same standardized approximately 250 g fried-rice meal. However, it was not calibrated to the habitual eating rates of the present sample or independently validated for this specific meal type. It should therefore be interpreted as a literature-informed prototype control parameter rather than a population- or meal-specific cutoff, participant-classification criterion, or behavioral outcome.

**Table 1:** SensEat feedback rules and output parameters.

Interaction elements		Eating rate ≤ 0.83 g/s	Eating rate > 0.83 g/s	Output parameter
Visual	LED timing	√	–	+1 LED lit/min
	LED breathing	–	√	20 LEDs
Auditory	Calming music	√	–	Repeat one
Haptic	Gentle vibration	–	√	5 s

√ indicates that the feedback channel was active; − indicates that it was inactive; “+1 LED lit/min” indicates that one additional LED was illuminated each minute; “Repeat One” indicates continuous repetition of the selected audio track; and “5 s” indicates the vibration duration.

When a rapid intake rate is detected, the LED strip is triggered to enter a breathing-light mode, using visual rhythm to gently guide the user to slow down. If the user fails to adjust in time, the music pauses and a subtle vibration is activated as a secondary reminder. Once the eating pace returns to normal, the light and music resume their regular rhythm. All these mechanisms employ non-verbal, directional, and multi-channel cues to ensure sufficient perceptual salience without being intrusive.

Building upon the above conceptual framework, an interdisciplinary co-design workshop was conducted to further validate the feasibility and contextual adaptability of the proposed design. Experts and users from the fields of HCI, food and health, product design, and cognitive psychology were invited to participate in prototype testing and discussion. The feedback indicated that most participants perceived the system as having significant advantages in fostering eating rhythm and enhancing dietary awareness, with a high potential for natural integration and user acceptance in real-world dining environments.

### 2.2 Development of the prototype

To explore the design concept in a real dining environment and assess the feasibility of providing eating-pace guidance with minimal disruption, we employed a rapid prototyping approach that enables the demonstration and user evaluation of our design concept without the need for full implementation of all technical components and data infrastructure (53). Through an iterative design process, we developed a prototype of the smart tableware system consisting of two core modules: a lunchbox kit and a flexible placemat. The lunchbox lid features a windowed structure that accommodates dedicated chopsticks and a spoon for convenient portability. A miniature speaker is embedded on the top of the lunchbox, while a vibration motor is installed at the bottom. At the center of the placemat, a high-precision weight sensor continuously monitors changes in food weight for real time feedback control.

The weight signal was processed in real time to activate the feedback rules but was not stored for subsequent statistical analysis. Neither the continuous sensor stream nor participant-level feedback-trigger events, including trigger counts and timestamps, were logged. Although the dining sessions were video-recorded, the recordings did not consistently capture the device state and all feedback modalities throughout each session; therefore, trigger events could not be reconstructed reliably from the videos. Consequently, no formal or informal participant-level trigger log was available, and feedback-trigger frequency could not be used as a manipulation check or behavioral outcome.

**Figure 4:**
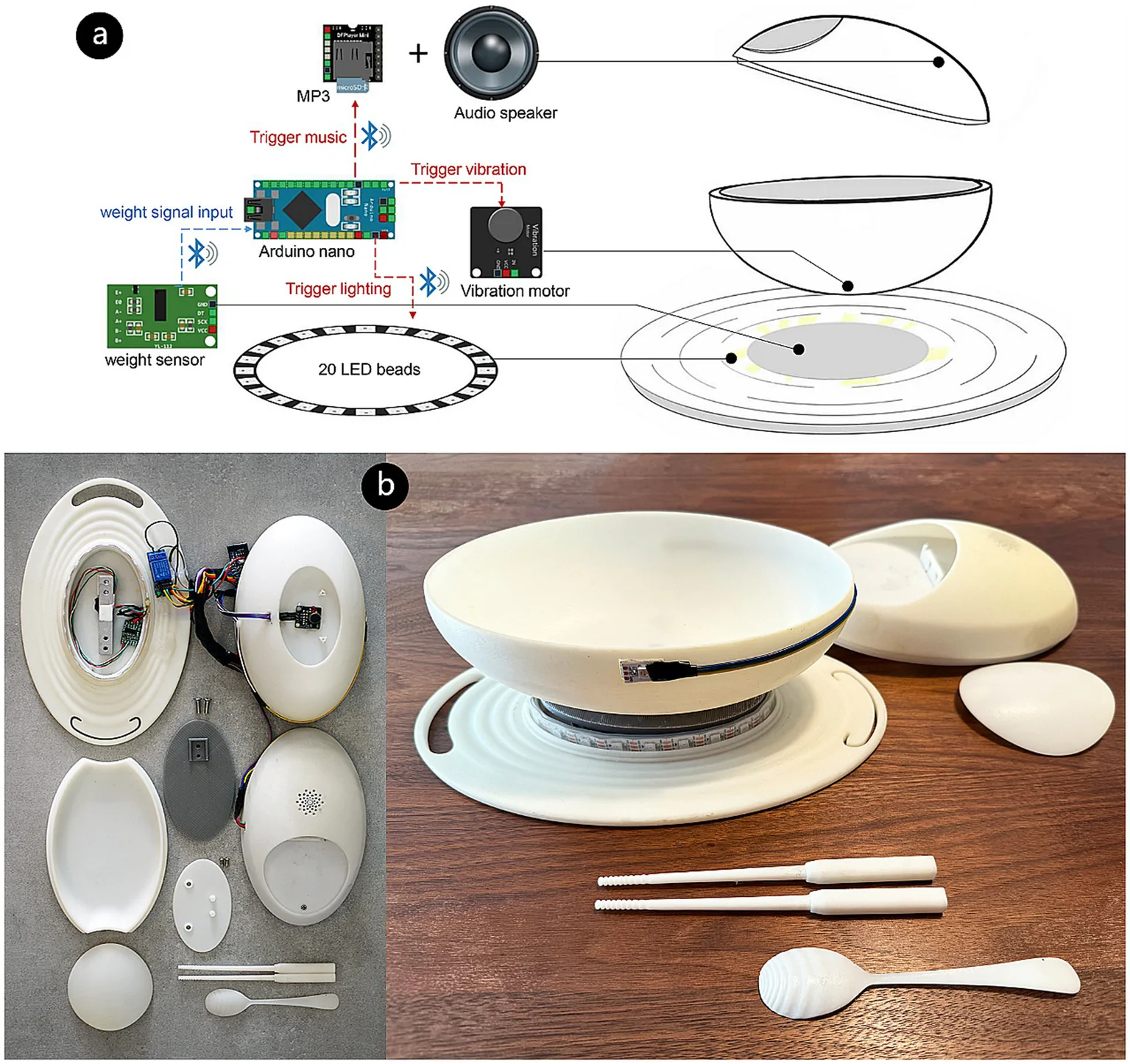
Development of prototype. **(a)** Prototypes of the sensors. **(b)** The prototype of the SensEat based on 3D printing technology and integration of smart hardware.

Twenty LED beads form a visual cue system around lunchbox’s perimeter for the “micro-task.” The underside of the placemat houses an integrated Arduino Nano mainboard, which synchronizes with other components via Bluetooth (Figure 4a). During the rapid prototyping phase, we used Rhino for structural modeling with a particular focus on optimizing the embedding space for electronic components and the routing of wiring. The lunchbox and tableware shells were fabricated from high-strength polylactic acid (PLA) material via 3D printing, while the placemat was produced from thermoplastic polyurethane (TPU) through 3D printing to preserve its inherent elasticity. All sensing modules were integrated and calibrated with an Arduino Nano control board, culminating in a fully functional prototype system that can be deployed in real-world dining environments (Figure 4b).

At the system level, the prototype instantiated a feedback loop comprising weight-change sensing, an operational intake-rate threshold, graded multimodal cueing, and return to the ambient state, with the interaction distributed across the tableware rather than presented on a separate screen.

### 2.3 Study design and experimental conditions

The SensEat system was employed as a technology probe and deployed across three solo-dining settings. The aim of this study was to explore within-participant differences in mindful-eating behaviors, self-reported experience, and perceived task workload between an app-guided intervention session and a SensEat-guided intervention session. All participants completed the tests individually with the task defined as consuming the same standardized meal. This pilot study used an exploratory fixed-sequence within-subject comparative design to compare an app-guided intervention (AGI) session with a SensEat-guided intervention (SGI) session. All participants completed the AGI session first, followed by the SGI session 3 days later. The fixed order was primarily a pragmatic procedural choice made to maintain a consistent workflow across participants and study settings, with the commercially available app condition administered before deployment of the SensEat prototype. It was not intended to establish a no-guidance baseline or to control sequence effects. No randomization or counterbalancing of session order was used.

In the SGI condition, participants used the SensEat system, which continuously monitored food consumption through an integrated weight sensor and delivered real-time multimodal feedback via ambient lighting, auditory cues, and haptic feedback throughout the meal. In the AGI condition, participants used regular tableware together with SlowEat (version 1.6.1 for iOS; developed by Ji, Kaicheng; Apple App Store ID: 6742094566), a commercially available smartphone-based mindful-eating application (54). The version was released on December 5, 2025 and introduced Smart Detection Mode, which used the smartphone camera to detect chewing speed and automatically delivered reminders when rapid eating was detected. During the experiment, only this real-time camera-based pacing-guidance function was enabled; long-term behavior tracking, achievement systems, and wearable-device functions were not used.

Accordingly, both sessions provided real-time behavioral guidance toward eating-pace regulation. However, the comparison was conducted at the system level: AGI and SGI differed not only in screen-based versus embodied interaction, but also in sensing mechanism, feedback modality, feedback dynamics, and the explicit 20-min pacing scaffold included in SGI. The study therefore did not isolate the independent effect of embodied interaction from these other system-level differences. Specifically, AGI relied on screen-based audio-visual feedback and computer-vision-based monitoring, whereas SGI embedded behavioral feedback directly into the tableware through multimodal embodied interaction. The main similarities and system-level differences between the AGI and SGI sessions are summarized in Table 2.

**Table 2:** System-level comparison of the AGI and SGI sessions.

Feature	AGI (SlowEat v1.6.1, iOS)	SGI (SensEat)
Intervention type	Screen-based mindful eating intervention	Embodied mindful eating intervention
Behavioral monitoring	Smartphone camera–based monitoring of chewing activity	Integrated weight sensor monitoring food-weight changes
Eating pace estimation	Camera-based detection of chewing speed	Food-consumption rate estimated from real-time weight changes
Feedback	Automatic visual and auditory reminders when rapid eating was detected	Real-time visual, auditory, and haptic cues
Interaction medium	Smartphone screen	Feedback embedded directly into the tableware
Dining utensil	Regular tableware	Smart tableware
Pacing scaffold	No equivalent LED-based pacing scaffold	20-min pacing scaffold with twenty 1-min LED units

Both sessions provided real-time guidance toward eating-pace regulation and used the same standardized meal. The comparison was conducted at the system level rather than isolating the independent effect of a single interaction feature. AGI, app-guided intervention; SGI, SensEat-guided intervention.

During the tests, two cameras were used to record the entire dining process. The two sessions were separated by a three-day interval, and all study sessions were completed within 1 week. The overall objective of this study was to compare within-participant differences in behavioral outcomes (RQ1), self-reported experience ratings (RQ2), and perceived task workload (RQ3) between the AGI and SGI sessions. Because the study used a fixed AGI → SGI sequence without counterbalancing, the analyses were intended to describe session-level within-participant differences rather than to estimate a causal condition effect independent of order.

### 2.4 Participants

Previously the effects of spatial layout and occupational context on eating behaviors and dining culture were investigated (55, 56). All participants were from Beijing Technology and Business University. Group A included six participants (2 females and 4 males, 20.89 ± 1.52 years old) from engineering and art disciplines at a design studio within a university. The dining area was located in an open-plan office comprising both formal workspaces and a break area. Participants dined individually at tables in the break area, using the SensEat system and consuming identical meals. In Group B, seven undergraduate and graduate students who habitually dined in the university cafeteria were recruited (4 females and 3 males, 23.28 ± 1.56 years old). They were familiar with the cafeteria environment and dined individually at vacant tables using SensEat with the same meal. Group C included seven participants (5 females and 2 males, 29.37 ± 6.23 years old) at a university apartment, including three early-career instructors and four students. They were accustomed to bringing food back to their apartments and dining alone. Participants were provided with the SensEat system and identical meals for the study. Participant demographics and study settings are summarized in Table 3. The three settings were included to provide contextual diversity rather than to support formal between-setting comparisons. Because the subgroup sizes were small (n = 6, 7, and 7), data from the three groups were pooled for the primary within-participant analyses. Potential effects associated with group, age, study setting, or occupational context could not be reliably estimated and were not formally tested.

**Table 3:** Participant demographics and study settings by group.

Group	Study setting	*n*	Age, mean ± SD, years	Sex, n (female/male)
A	Design studio within a university	6	20.89 ± 1.52	2/4
B	University cafeteria	7	23.28 ± 1.56	4/3
C	University apartment	7	29.37 ± 6.23	5/2

Age is presented as mean ± standard deviation. Sex is presented as female/male counts. Because of the small subgroup sizes, no formal tests of between-group heterogeneity or moderation by age or study setting were conducted.

### 2.5 Procedure

Prior to the study, all participants were briefed on the experimental procedure and signed an informed consent form. Their schedules were confirmed to ensure sufficient time for participation and to avoid rushed eating behaviors due to time pressure. To minimize the influence of food characteristics on eating behavior, all participants were provided with the same standardized meal in both intervention sessions. The meal consisted of approximately 250 g of fried rice (rice stir-fried with vegetables), prepared by the same university cafeteria. Identical meals were served across all three dining settings and both intervention sessions. Although meal type, serving mass, and food source were standardized, the energy content and energy density of the meals were not independently quantified.

**Figure 5:**
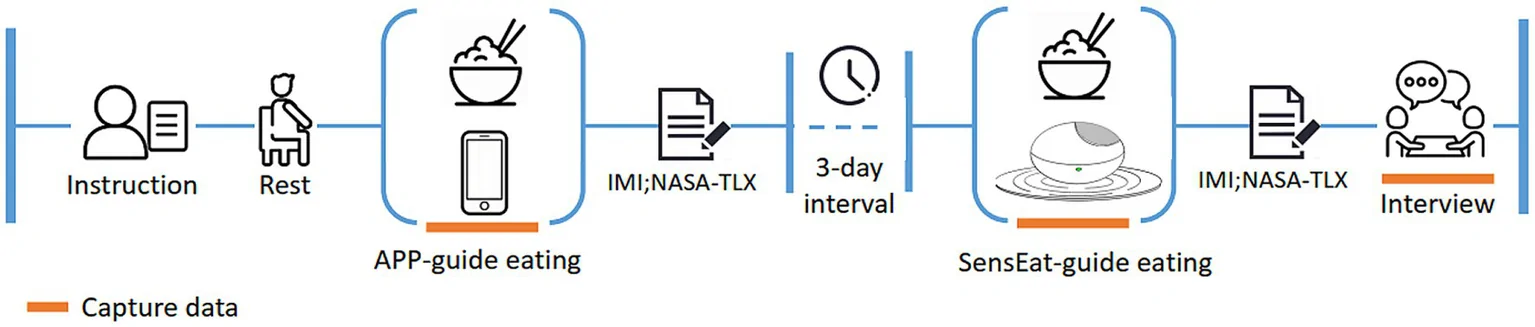
Experimental procedure.

**Figure 6:**
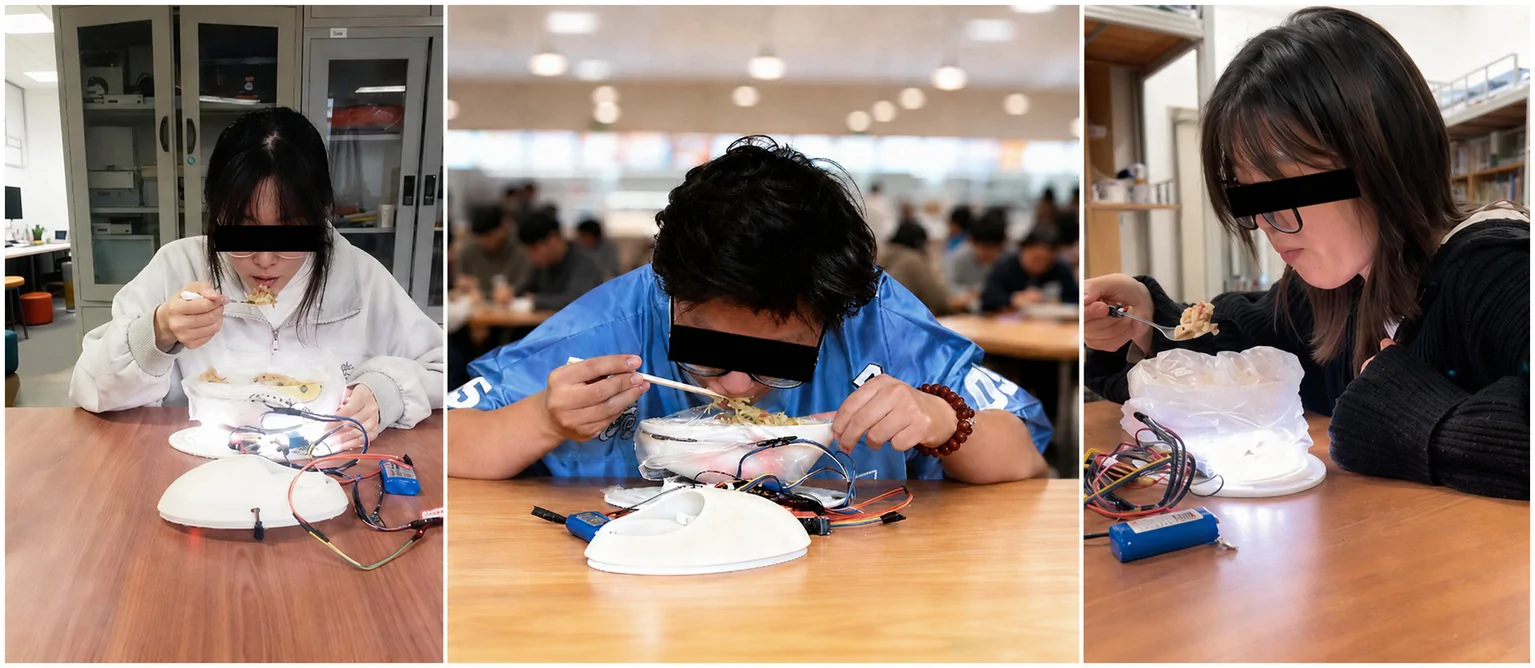
The participants were experiencing SensEat.

During the experiment, each participant completed two intervention sessions using the same standardized meal (Figure 5). With participants’ consent, the entire dining process was recorded by two cameras to capture detailed behavioral data. The participants first completed the AGI using SlowEat version 1.6.1 on the iOS platform, which provided camera-based detection of chewing speed together with real-time visual and auditory reminders during the meal. All formal AGI–SGI comparative sessions analyzed in this study were conducted after version 1.6.1 and its Smart Detection Mode had become available. Immediately after the AGI session, participants completed five IMI-informed single-item ratings and three selected NASA-TLX ratings. After a three-day interval, the participants dined at the same time of day using the SensEat system (Figure 6). Immediately after the SGI session, they completed the same self-report measures and then took part in a single semi-structured interview. The interview focused on their experience with SensEat and also included comparisons between the AGI and SGI sessions. The study concluded after the participants completed both experimental sessions.

### 2.6 Measurement

Both quantitative and qualitative data were collected from participants to evaluate behavioral performance and psychological experiences associated with our design. To identify behavioral changes across conditions (RQ1), video recordings were manually analyzed to extract and compare each participant’s meal duration and chews per bite. Meal duration was defined as the interval between the first bite and the completion of the final swallow, whereas chews per bite was defined as the number of chewing cycles occurring between two consecutive bites. For each session, chews per bite was calculated for every observed bite and then averaged to obtain one participant-level value for statistical analysis. All video recordings were initially coded by the first author following a predefined coding protocol. To assess inter-rater reliability, a second researcher independently re-coded both session recordings from four randomly selected participants, representing 8 of the 40 recordings (20%). Inter-rater reliability was evaluated separately for meal duration and mean chews per bite using two-way mixed-effects, absolute-agreement, single-measure intraclass correlation coefficients with 95% confidence intervals. The point estimates indicated high inter-rater reliability for meal duration [ICC = 0.96, 95% CI (0.81, 0.99)] and chews per bite [ICC = 0.92, 95% CI (0.66, 0.98)]. Because the intervention device was clearly visible in the video recordings, blinding coders to the experimental condition was not feasible.

To assess self-reported experience (RQ2), five study-specific single-item ratings were used, informed by the IMI constructs of Interest/Enjoyment, Value/Usefulness, Perceived Competence, Effort/Importance, and Pressure/Tension (57). Each construct was represented by one positively keyed item rated on a 7-point scale. No reverse scoring, multi-item aggregation, or overall IMI score was used. Accordingly, these measures are described as IMI-informed single-item ratings rather than standard multi-item IMI subscales. Because each construct was assessed using a single item, within-construct internal consistency could not be estimated.

To assess perceived workload (RQ3), three selected NASA-TLX dimensions—Mental Demand, Performance, and Frustration—were each assessed using a separate single-item rating (58). These ratings were analyzed separately, and no overall NASA-TLX workload score was calculated. Because each selected dimension was represented by a single rating and the three dimensions were not treated as a unidimensional scale, an internal-consistency coefficient was not applicable. Mental Demand and Frustration were rated on 20-point scales, with higher scores indicating greater perceived demand or frustration. For the Performance rating, we used adapted response anchors ranging from 1 (“poor performance”) to 20 (“good performance”); therefore, higher Performance scores indicated better perceived performance. This direction differed from the original NASA-TLX Performance scale, which is conventionally anchored from good to poor. The adapted scoring direction was used consistently in both experimental sessions and in all statistical analyses. Immediately after completing the post-SGI questionnaires, each participant took part in a semi-structured interview lasting approximately 30 min. The interview focused primarily on participants’ experience with SensEat and also invited them to compare the AGI and SGI sessions and explain their preferences. Example questions included “Please describe your experience while dining with SensEat,” “Which dining system did you prefer, SlowEat or SensEat, and why?,” “How would you evaluate SensEat overall?,” and “What suggestions do you have for improving the system?” Sufficient time was provided for participants to freely express their experiences and reflections. All interviews were audio-recorded and transcribed verbatim for subsequent qualitative analysis.

### 2.7 Data analysis

#### 2.7.1 Quantitative data

All quantitative analyses were conducted using IBM SPSS Statistics, version 27.0.1.0 (IBM Corp., Armonk, NY, United States). All 20 participants provided complete paired data for all outcomes; no imputation was performed. Normality was assessed for the within-participant difference scores (SGI − AGI) for each outcome using the Shapiro–Wilk test. Paired-samples *t*-tests were used for chews per bite, Perceived Competence, Pressure/Tension, Performance, and Frustration. Wilcoxon signed-rank tests were used for meal duration, Interest/Enjoyment, Effort/Importance, Value/Usefulness, and Mental Demand. All tests were two-sided. Eating rate in g/s and feedback-trigger frequency were not included as outcomes because neither the continuous weight-sensor stream nor participant-level trigger events were logged for offline analysis.

To control the family-wise error rate, the Holm correction was applied separately to the behavioral outcomes (2 comparisons), the five IMI-informed single-item ratings (5 comparisons), and the three selected NASA-TLX ratings (3 comparisons). Both unadjusted and P_Holm_ values were reported. Exact *p* values were provided except for values below 0.001, which were reported as *p* < 0.001. Statistical significance was evaluated using P_Holm_ < 0.05.

For paired-samples *t*-tests, results were reported as mean ± SD, t(df), unadjusted p, P_Holm_, Cohen’s *d_z_* with its two-sided 95% confidence interval, and the mean paired difference (SGI − AGI) with its 95% confidence interval. Confidence intervals for Cohen’s *d_z_* were calculated by inversion of the noncentral t distribution.

For Wilcoxon signed-rank tests, results were reported as median [IQR], z, two-sided asymptotic p, P_Holm_, and the matched-pairs rank-biserial correlation (r_rb_) with its two-sided 95% confidence interval. The matched-pairs rank-biserial correlation was calculated as (W^+^ − W^−^)/(W^+^ + W^−^), where W^+^ and W^−^ represent the sums of positive and negative signed ranks, respectively, based on the paired differences (SGI − AGI). Zero differences were excluded, and tied absolute differences were assigned average ranks. Positive r_rb_ values indicated higher observations in the SGI session. Percentile-bootstrap 95% confidence intervals for r_rb_ were estimated by participant-level resampling of the paired observations with 100,000 resamples. The confidence intervals were not adjusted for multiple comparisons; statistical significance was evaluated using the Holm-adjusted *p* values. A reference sensitivity analysis based on a two-sided paired-samples *t*-test with *n* = 20, *α* = 0.05, and 80% power indicated a minimum detectable paired effect of *d_z_* = 0.66.

#### 2.7.2 Qualitative data

All interview transcripts were analyzed using a team-based inductive thematic analysis approach supported by affinity diagramming. The first author conducted the initial coding of all transcripts and organized related codes into candidate themes. A second researcher reviewed the coding framework and candidate-theme structure, and differences in interpretation were discussed until an agreed thematic organization was reached. Because the first author was also involved in the design of SensEat, the second researcher’s review of the coding framework and divergent cases was used to reduce reliance on single-researcher interpretation. This process was used to enhance analytic credibility and reflexive discussion rather than to calculate formal inter-coder reliability. The themes were iteratively refined, with attention to both recurring patterns and divergent participant experiences. For descriptive transparency, theme prevalence was summarized as the number of unique participants who raised each theme at least once. Repeated references to the same theme by one participant were counted once. These counts describe the occurrence of themes within this sample and were not used for statistical generalization.

## 3 Results

**Figure 7:**
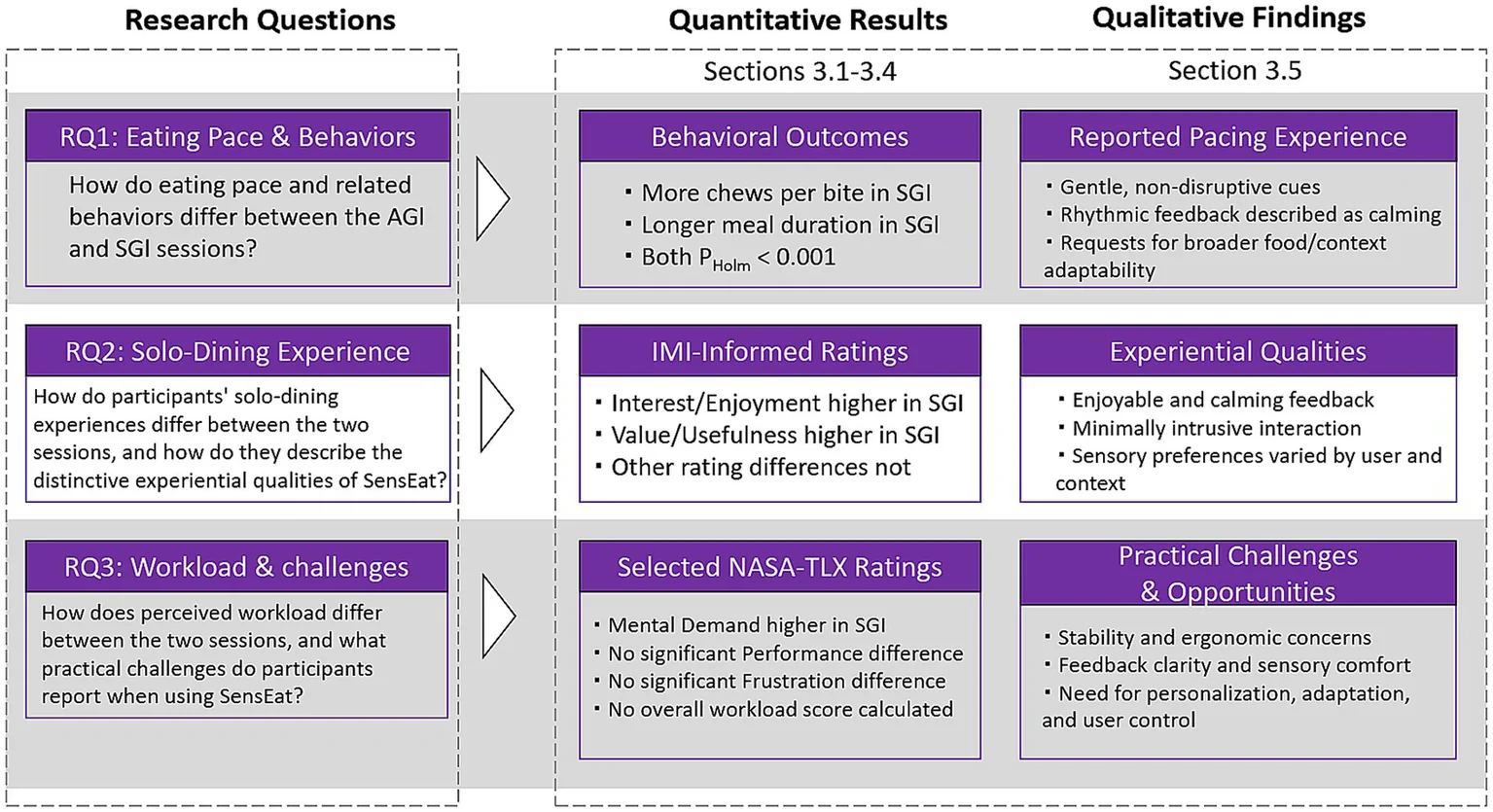
Alignment of the research questions with the quantitative results and qualitative findings.

Both the quantitative and qualitative data were analyzed to address the three research questions (Figure 7).

### 3.1 Behavioral differences between the SGI and AGI sessions

**Figure 8:**
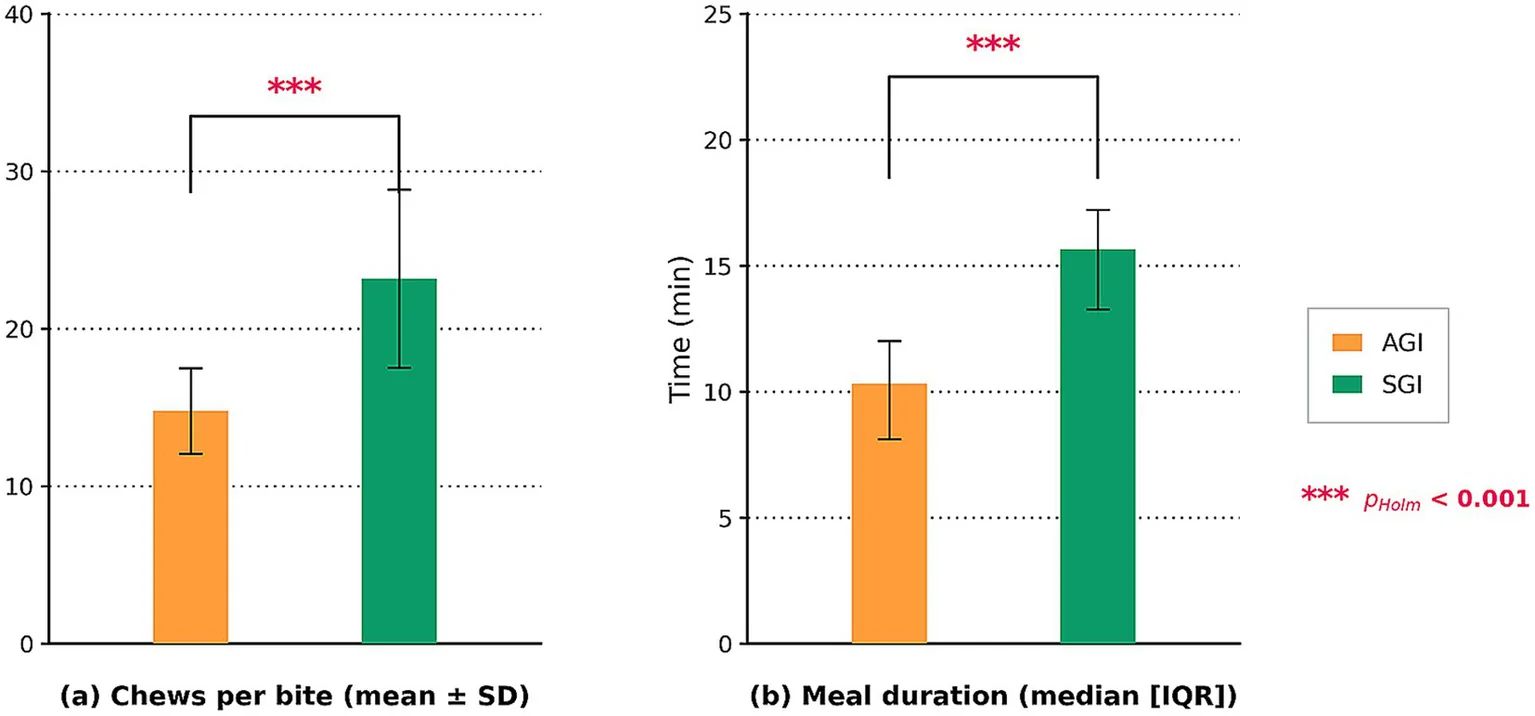
Behavioral outcomes in the AGI and SGI sessions: **(a)** chews per bite, presented as mean ± SD; and **(b)** meal duration, presented as median and IQR. Significance annotations are based on Holm-adjusted *P* values.

Chews per bite and meal duration were compared between the AGI and SGI sessions (Figure 8; individual paired observations are shown in Supplementary Figure S1). Chews per bite were higher in the SGI session (23.17 ± 5.66 chews/bite) than in the AGI session (14.77 ± 2.72 chews/bite), *t* (19) = 8.05, *p* < 0.001, P_Holm_ < 0.001, Cohen’s *d_z_* = 1.80, 95% CI (1.07, 2.51), with a mean paired difference of 8.41 chews per bite, 95% CI (6.22, 10.59) (Figure 8a). Meal duration was also longer in the SGI session (Mdn = 15.65 min, IQR = 13.27–17.23) than in the AGI session (Mdn = 10.33 min, IQR = 8.12–12.01), z = −3.92, *p* < 0.001, P_Holm_ < 0.001, r_rb_ = 1.00, 95% CI (1.00, 1.00) (Figure 8b). At the participant level, meal duration was longer in the SGI session for all 20 participants, and chews per bite were higher for 19 of 20 participants. These findings were consistent with a slower eating pattern in the SGI session than in the preceding AGI session. However, because all participants completed AGI before SGI, these session-level differences cannot be attributed solely to the intervention condition.

### 3.2 Differences in self-reported experience between the AGI and SGI sessions

**Figure 9:**
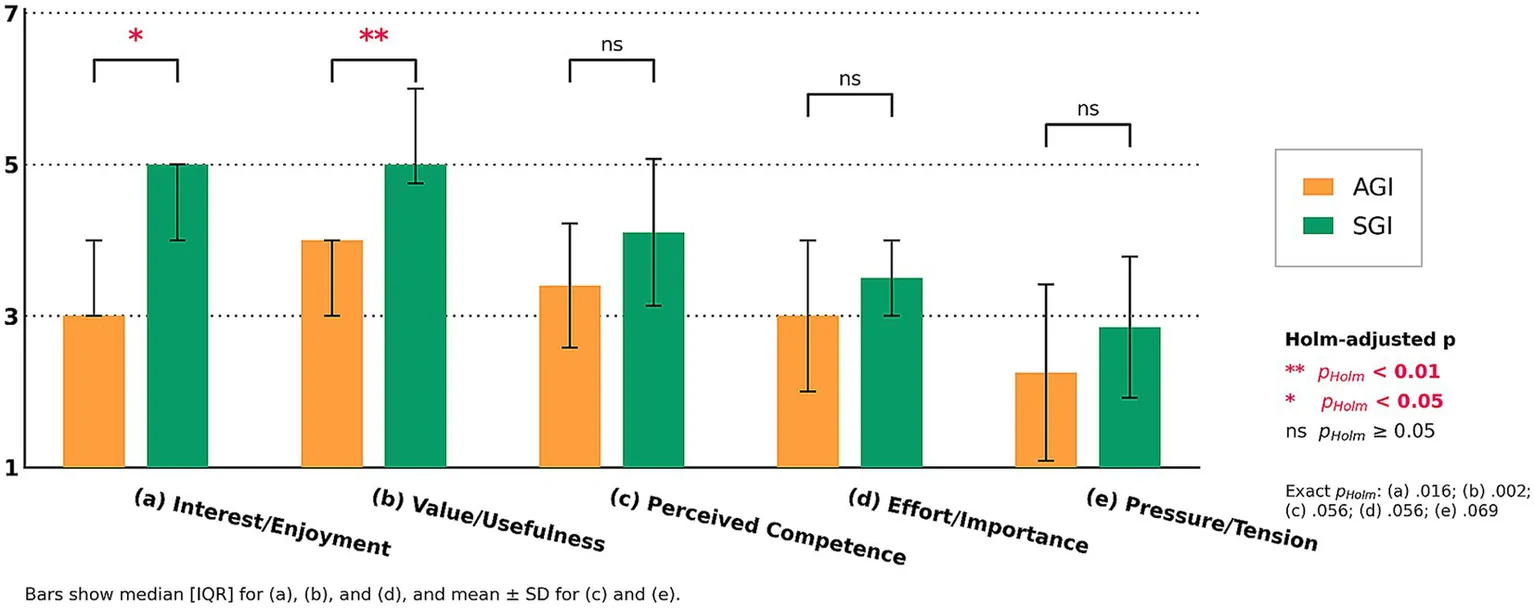
IMI-informed single-item ratings in the AGI and SGI sessions: **(a)** Interest/Enjoyment; **(b)** Value/Usefulness; **(c)** Perceived Competence; **(d)** Effort/Importance; and **(e)** Pressure/Tension. Outcomes analyzed using Wilcoxon signed-rank tests (panels **a**, **b**, and **d**) are presented as median and IQR; outcomes analyzed using paired-samples *t*-tests (panels **c** and **e**) are presented as mean ± SD. Significance annotations are based on Holm-adjusted *P* values.

Five IMI-informed single-item ratings were compared between the AGI and SGI sessions (Figure 9; individual paired observations are shown in Supplementary Figure S2). After Holm correction, statistically significant differences remained only for Interest/Enjoyment and Value/Usefulness. Interest/Enjoyment was higher in the SGI session (Mdn = 5.00, IQR = 4.00–5.00) than in the AGI session (Mdn = 3.00, IQR = 3.00–4.00), *z* = −2.88, *p* = 0.004, P_Holm_ = 0.016, r_rb_ = 0.83, 95% CI (0.44, 1.00) (Figure 9a). Value/Usefulness was also higher in the SGI session (Mdn = 5.00, IQR = 4.75–6.00) than in the AGI session (Mdn = 4.00, IQR = 3.00–4.00), *z* = −3.57, *p* < 0.001, P_Holm_ = 0.002, r_rb_ = 0.96, 95% CI (0.82, 1.00) (Figure 9b).

The remaining three single-item ratings did not differ significantly after Holm correction. Before multiplicity correction, Perceived Competence was higher in the SGI session (4.10 ± 0.97) than in the AGI session (3.40 ± 0.82), *t* (19) = 2.57, *p* = 0.019, P_Holm_ = 0.056, Cohen’s *d_z_* = 0.57, 95% CI (0.09, 1.04), with a mean paired difference of 0.70, 95% CI (0.13, 1.27) (Figure 9c). Effort/Importance was Mdn = 3.50 (IQR = 3.00–4.00) in the SGI session and Mdn = 3.00 (IQR = 2.00–4.00) in the AGI session, *z* = −2.24, *p* = 0.025, P_Holm_ = 0.056, r_rb_ = 0.63, 95% CI (0.18, 1.00) (Figure 9d). Pressure/Tension was 2.85 ± 0.93 in the SGI session and 2.25 ± 1.16 in the AGI session, *t* (19) = 1.93, *p* = 0.069, P_Holm_ = 0.069, Cohen’s *d_z_* = 0.43, 95% CI (−0.03, 0.88), with a mean paired difference of 0.60, 95% CI (−0.05, 1.25) (Figure 9e).

Because all participants completed AGI before SGI, these results represent self-reported session-level differences and cannot be attributed solely to the intervention condition.

### 3.3 Perceived workload differences between the SGI and AGI sessions

**Figure 10:**
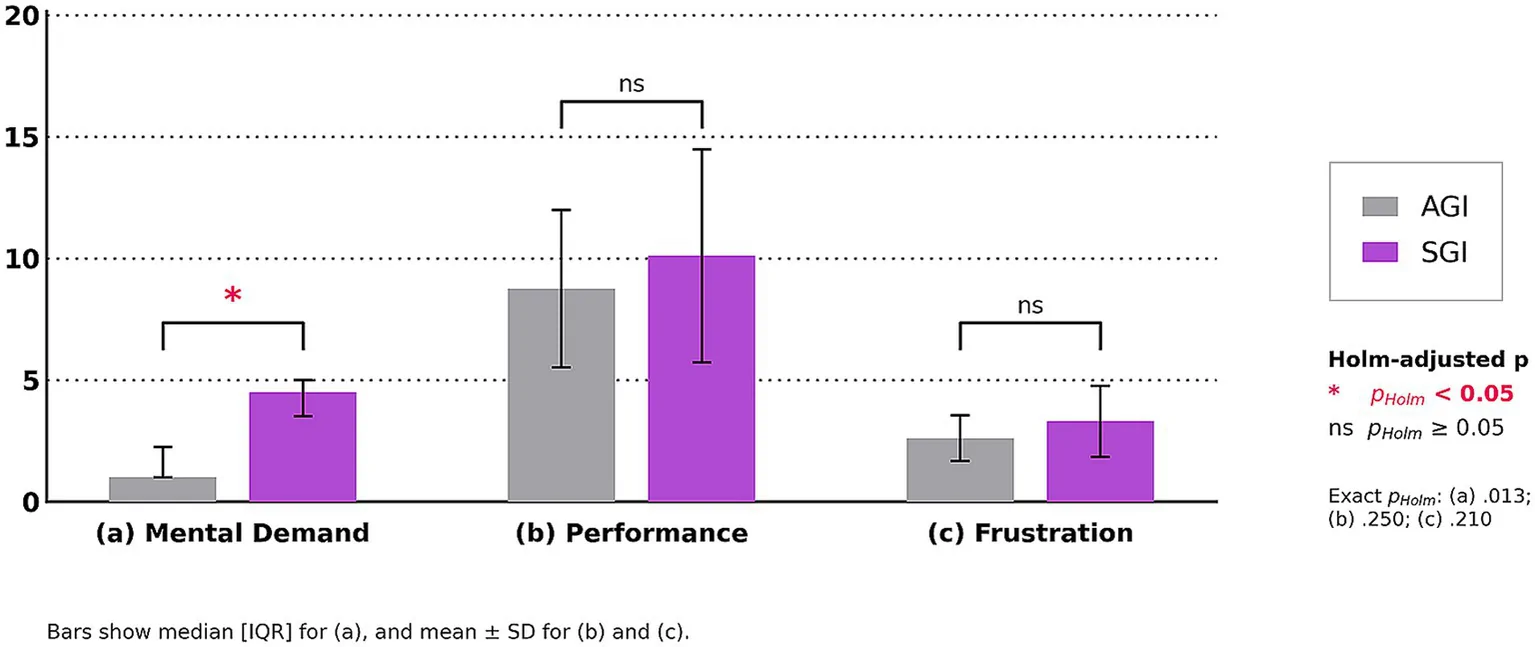
Selected NASA-TLX ratings in the AGI and SGI sessions: **(a)** Mental Demand, presented as median and IQR; **(b)** Performance, presented as mean ± SD; and **(c)** Frustration, presented as mean ± SD. Significance annotations are based on Holm-adjusted *P* values. For the adapted Performance rating, higher scores indicate better perceived performance; for Mental Demand and Frustration, higher scores indicate greater perceived demand or frustration.

Three selected NASA-TLX ratings were compared between the AGI and SGI sessions (Figure 10; individual paired observations are shown in Supplementary Figure S3). The ratings were analyzed separately, and no overall NASA-TLX workload score was calculated. Mental Demand was higher in the SGI session (Mdn = 4.50, IQR = 3.50–5.00) than in the AGI session (Mdn = 1.00, IQR = 1.00–2.25), *z* = −2.85, *p* = 0.004, P_Holm_ = 0.013, r_rb_ = 0.75, 95% CI (0.39, 0.98) (Figure 10a).

Under the adapted scoring direction used in this study, higher Performance scores indicated better perceived performance. Performance did not differ significantly between the SGI session (10.10 ± 4.38) and the AGI session (8.75 ± 3.24), *t* (19) = 1.19, *p* = 0.250, P_Holm_ = 0.250, Cohen’s *d_z_* = 0.27, 95% CI (−0.18, 0.71), with a mean paired difference of 1.35, 95% CI (−1.03, 3.73) (Figure 10b). Frustration also did not differ significantly between the SGI session (3.30 ± 1.45) and the AGI session (2.60 ± 0.94), *t* (19) = 1.70, *p* = 0.105, P_Holm_ = 0.210, Cohen’s *d_z_* = 0.38, 95% CI (−0.08, 0.83), with a mean paired difference of 0.70, 95% CI (−0.16, 1.56) (Figure 10c).

Thus, Mental Demand was higher in the SGI session, whereas no statistically significant session differences were found for Performance or Frustration after Holm correction. The nonsignificant findings should not be interpreted as evidence of equivalence or the absence of individual differences. Given the fixed AGI → SGI sequence, these findings describe session-level differences and cannot separate intervention effects from order-related effects.

### 3.4 Summary of quantitative findings

As evidenced by the longer meal duration and greater number of chews per bite, participants exhibited a slower eating pace in the SGI session than in the AGI session (RQ1). Both behavioral differences remained statistically significant after Holm correction. These findings describe differences in eating behavior between the two sessions but do not directly establish that SensEat improved participants’ self-regulation or increased their attention to eating pace. As noted by O’Reilly et al. (59), an effective approach to improving eating behavior is to encourage individuals to focus on both the food and their own eating actions during meals. Although attention and self-regulation were not directly measured in the present study, the observed differences in eating pace provide a basis for further investigating these potential mechanisms.

The IMI-informed single-item ratings also revealed differences in participants’ self-reported experiences between the SGI and AGI sessions (RQ2). Specifically, participants reported significantly higher Interest/Enjoyment and Value/Usefulness in the SGI session. Although the unadjusted comparisons for Perceived Competence and Effort/Importance were statistically significant, neither difference remained significant after Holm correction. Pressure/Tension also did not differ significantly between the sessions. Therefore, the quantitative evidence for experiential differences was limited to higher Interest/Enjoyment and Value/Usefulness ratings in the SGI session.

Furthermore, the selected NASA-TLX ratings reflected participants’ perceived workload during the two sessions (RQ3). Mental Demand was significantly higher in the SGI session than in the AGI session. In contrast, no statistically significant differences were found for Performance or Frustration. Because only three NASA-TLX dimensions were assessed separately and no overall workload score or acceptability threshold was used, these results should not be interpreted as demonstrating an acceptable overall cognitive workload. Moreover, higher Mental Demand indicates greater perceived task demand but does not, by itself, demonstrate greater attentional engagement.

Taken together, these findings describe session-level differences in eating pace, Interest/Enjoyment, Value/Usefulness, and Mental Demand. Because attention and mindful awareness were not directly measured, the results should not be interpreted as evidence that SensEat increased these constructs.

### 3.5 Interview results

As described in section 2.7.2, the interview data were analyzed using inductive thematic analysis. Detailed qualitative findings organized according to RQ1–RQ3 are provided in Supplementary material; this section summarizes the principal cross-cutting themes. Participants generally described SensEat’s embodied multimodal feedback as enjoyable, relatively unobtrusive, and supportive of eating-pace awareness. In particular, they appreciated how the rhythmic cues were integrated into the dining artifacts rather than delivered through a smartphone screen. As P15 explained, “The breathing-like light rhythm really guided my chewing speed. At first, it was a bit tense, but soon it became calming.”

Participants also proposed possible extensions, including support for different food types, gamification, and post-meal behavioral summaries. These suggestions represent future design opportunities rather than functions evaluated in the present study.

Participants also identified practical limitations related to physical ergonomics and variability in the perception of sensory cues. For example, P11 noted, “The bowl sometimes slides on the mat, so I have to hold it while eating.” Although many participants appreciated the multimodal feedback, some found cues repetitive, weak, or intrusive in certain dining contexts. P14 commented, “I like the light feedback, but the sound feels a bit intrusive when I eat alone at night.” These contrasting preferences highlight the need for greater user control and personalization, such as adjusting cue intensity, selecting or muting individual feedback modalities, and adapting feedback to individual eating patterns and dining contexts. The principal perceived benefits, practical challenges, and proposed design opportunities are summarized in Table 4.

**Table 4:** Summary of qualitative themes and participant counts for SensEat.

System	Benefits	Challenges
SensEat	• Sensory-aesthetic immersion (*n* = 14, 70%)	• Stability and ergonomic issues (*n* = 6, 30%)
	• Companion-like comfort (*n* = 8, 40%)	• Variable feedback clarity and sensory comfort (*n* = 11, 55%)
	• Shared rhythm/co-presence (*n* = 5, 25%)	• Need for personalization and user control (*n* = 9, 45%)

n indicates the number of unique participants, out of 20, who raised each theme at least once. Repeated references to the same theme by one participant were counted once. Participants could contribute to more than one theme; therefore, the counts are not mutually exclusive and do not sum to 20. Shared rhythm/co-presence was a subtheme of companion-like comfort. The counts are descriptive of this sample and should not be interpreted as estimates of population prevalence.

## 4 Discussion

Mindful eating has been widely examined as a behavioral approach for supporting dietary regulation and addressing modifiable behavioral risk factors associated with chronic non-communicable diseases (NCDs). This study proposed and evaluated SensEat, a multimodal embodied smart tableware system designed to provide contextually embedded feedback intended to encourage attention to eating pace in solo dining contexts through implicit micro-feedback. Based on an exploratory fixed-sequence within-subject comparison, our results showed that participants had longer meal duration and significantly more chews per bite in the SGI session than in the AGI session, with both differences remaining significant after Holm correction (RQ1). These session-level differences were consistent with a slower eating pace during the SGI session. Participants also reported higher Interest/Enjoyment and Value/Usefulness in the SGI session (RQ2). However, the differences in Perceived Competence and Effort/Importance did not remain statistically significant after Holm correction, and Pressure/Tension did not differ significantly between the sessions. Thus, the quantitative evidence for experiential differences was limited to Interest/Enjoyment and Value/Usefulness rather than reflecting a general improvement across all five self-reported experience ratings. Qualitative results further revealed that participants described the multisensory feedback, including light, sound, and haptic cues, as companion-like, calming, and supportive, and some participants reported that these cues helped them remain attentive to the dining process.

Although Effort/Importance was higher in the SGI session before multiplicity correction, this difference did not remain statistically significant after Holm correction. Its directional increase may reflect greater motivational involvement, but it may also indicate that participants perceived the SensEat interaction as requiring more effort, particularly when considered alongside the higher Mental Demand score. This interpretation therefore remains tentative.

The selected NASA-TLX ratings showed that Mental Demand was higher in the SGI session, whereas Performance and Frustration did not differ significantly between the sessions after Holm correction (RQ3). Because only three NASA-TLX dimensions were analyzed and no overall workload score or predefined acceptability threshold was used, the quantitative results do not establish that the overall cognitive workload was low or acceptable. Qualitative feedback suggested that many participants considered the interaction itself acceptable during the short experimental sessions. However, this qualitative perception should not be interpreted as quantitative evidence that the overall workload was low or acceptable.

Taken together, the simultaneous increases in Interest/Enjoyment, Value/Usefulness, and Mental Demand highlight an important design tension. Screen-independent and artifact-embedded guidance should not be assumed to be cognitively effortless: the perceptible multimodal cues that may enhance experiential salience may also require additional attention. This finding suggests that future HFI systems should treat modality selection, cue intensity, and timing as adaptive design parameters rather than fixed features.

Overall, integrating multimodal embodied interaction into everyday tableware was associated with behavioral patterns consistent with slower eating, as well as higher reported Interest/Enjoyment and Value/Usefulness, in solo dining contexts. However, attention, mindful eating, and behavioral self-regulation were not directly measured as independent outcomes. Moreover, because the AGI session always preceded the SGI session, the observed differences cannot be separated from possible order, learning, or repeated-exposure effects. The findings should therefore be interpreted as exploratory session-level associations rather than definitive causal effects of SensEat.

From a broader perspective, embedding behavioral cues directly into everyday dining artifacts may represent a promising approach for integrating health-related guidance into routine activities while reducing reliance on continuous screen-based interaction. At the same time, the higher Mental Demand reported in the SGI session indicates that the cognitive implications of artifact-mediated guidance require further consideration.

Such artifact-mediated interventions are conceptually consistent with sustainability-oriented health promotion, which emphasizes maintainable and contextually integrated lifestyle support. However, the maintainability and long-term cognitive demands of SensEat were not evaluated in the present short-term deployment and should be examined through longer-term studies. While the present findings are limited to short-term behavioral outcomes, they suggest potential pathways through which embodied interaction design could inform the design of technologies intended to support healthier daily practices and wellbeing. These characteristics may be conceptually relevant to the preventive health priorities reflected in SDG 3 (Good Health and Wellbeing), particularly in relation to preventive lifestyle interventions and behavioral self-regulation. On the basis of these exploratory findings, three design implications are stated below for future Human–Food Interaction (HFI) systems and behavioral health interventions.

### 4.1 Supporting eating-pace regulation through implicit multimodal feedback

Our findings suggest that eating behavior differed between the two system-level sessions: the SGI session was associated with longer meal duration and more chews per bite than the preceding AGI session (Figure 8). Participants emphasized that these cues were “gentle reminders rather than commands,” suggesting that they experienced the feedback as minimally intrusive (60). Unlike screen-based interventions that rely on explicit notifications, *SensEat* operates as an *implicit embodied guide*, embedding feedback directly into the dining action. Participants also described the interaction as “non-disruptive” and “part of the meal itself.” These qualitative accounts can be considered in relation to previous research on mindfulness-based eating interventions (61) and multisensory integration during eating (62). However, these studies did not directly evaluate a SensEat-like tableware system, and the present findings do not establish reduced cognitive overload or increased mindful awareness.

Future HFI systems may thus explore adaptive feedback mechanisms that dynamically tune intensity or modality based on individual eating patterns or emotional states (63). Such adaptation may be particularly important for balancing the perceptibility of feedback against the additional Mental Demand associated with interacting with the system. By embedding guidance within the natural flow of eating behavior rather than imposing external control, implicit embodied feedback may support eating-pace adjustment in everyday contexts. Such integration into routine activities may provide a promising design direction for behavioral health technologies that aim to support eating awareness while minimizing disruption to the dining experience. However, longitudinal and counterbalanced studies are needed to determine whether these short-term session-level differences translate into sustained behavioral change, improved health outcomes, or acceptable long-term use.

### 4.2 Participants’ perceptions of companionship during SensEat use

Beyond behavioral regulation, eight of 20 participants described their experience with SensEat as companion-like and emotionally comforting during solo dining. Participants described the system as “a quiet dining partner” and “a companion that shared the moment,” suggesting that some users interpreted the embodied feedback in social or companion-like terms. This finding resonates with Ruddock and Brunstrom’s (2021) concept of social facilitation in eating, which posits that the social context of meals strongly affects pace and enjoyment (64). Our findings suggest that certain aspects of this concept may also be relevant when embodied interaction is incorporated into solo dining experiences.

Within this broader companionship theme, five participants specifically referred to a sense of shared rhythm, responsiveness, or co-presence while interacting with SensEat. These accounts are conceptually related to Yu’s notion of cross-modal sociality, in which multisensory environmental cues may contribute to perceived social connectedness (65). Participants reported that these embodied cues made the system feel “responsive” and “emotionally attuned,” which is consistent with previous studies suggesting that tactile and ambient feedback can contribute to perceptions of companionship and emotional comfort in solitary contexts (66).

Separately, three participants suggested that SensEat might be extended to support remote shared dining experiences. This suggestion was not evaluated in the present study but is conceptually related to research on digital social proxies and may inform future HFI studies of remote shared dining (67). These findings represent participants’ short-term subjective interpretations of SensEat rather than evidence that the system created an actual social relationship or reduced loneliness. Remote shared dining and virtual co-dining were not evaluated and are presented only as directions for future research.

### 4.3 Participants’ experiences of sensory-aesthetic design

Our qualitative findings suggest a potential role for sensory aesthetics and physical form design in shaping participants’ short-term dining experiences. Fourteen of 20 participants described the tactile texture of the SensEat utensils and mats (e.g., the “grain-like” bowl and “ripple-patterned” mat) as creating a calming and immersive dining atmosphere that encouraged focus and enjoyment.

Similarly, Tao et al. argued that sensory resonance and playful aesthetics can enhance users’ engagement with everyday health-related activities by transforming daily health behaviors into emotionally rewarding experiences (68). Likewise, research in affective computing and HFI suggests that aesthetically attuned physical interactions can positively influence users’ affective experiences during routine activities (69). The aesthetic coherence of SensEat’s visual, tactile, and auditory cues was perceived by participants as supporting sustained attention without causing excessive distraction, echoing previous findings in which multisensory balance supports positive user experiences during interactive activities (70).

Alongside these positive accounts, 11 participants raised concerns about feedback clarity or sensory comfort, 9 requested greater personalization and user control, and 6 reported stability or ergonomic issues.

Taken together, these findings suggest that embedding sensory aesthetics directly into the dining process may enrich participants’ affective experiences, although feedback should be adapted to individual preferences and practical contexts. These experiential accounts suggest that sensory-aesthetic qualities may offer useful design considerations for future HFI systems. Further studies should examine how adaptive sensory feedback can accommodate individual preferences and dining contexts without increasing distraction or perceived workload.

### 4.4 Limitations

Although several findings were statistically significant, several limitations should be acknowledged.

First, the study used a fixed AGI → SGI sequence without counterbalancing. As described in section 2.3, this order was a pragmatic procedural choice rather than a methodological strategy for controlling sequence effects. Consequently, intervention condition and session order were fully confounded, and order, novelty, expectancy, and repeated-exposure effects cannot be excluded. The observed differences therefore cannot be attributed solely to SensEat. The findings should therefore be interpreted as short-term, session-level associations and exploratory evidence regarding the feasibility and user experience of SensEat, rather than definitive evidence of intervention efficacy. Future studies should employ randomized counterbalanced designs. In addition, AGI and SGI were compared as whole systems and differed in interaction modality, sensing mechanism, feedback dynamics, and pacing structure, including the explicit 20-min pacing scaffold in SGI. The contribution of individual components could therefore not be isolated, and the longer meal duration observed during SGI may have been partly related to this pacing scaffold.

A related limitation concerns the qualitative data. The single semi-structured interview was conducted immediately after the SGI session and included retrospective comparisons with the AGI session, which had been completed 3 days earlier. These comparisons may therefore have been influenced by recall bias and by the greater recency or salience of the SGI experience. This concern applies primarily to the qualitative cross-session comparisons; the IMI and NASA-TLX measures were completed immediately after each respective session.

Second, a related implementation-fidelity limitation concerns the threshold-triggered feedback subsystem. The fixed threshold of >0.83 g/s (50 g/min) was selected as a literature-informed operational parameter but was not calibrated to participants’ habitual eating rates or independently validated for the standardized approximately 250 g fried-rice meal. Moreover, neither the continuous weight-sensor stream nor participant-level trigger events, including counts and timestamps, were logged. Because the video recordings did not consistently capture the device state and all feedback modalities, trigger events could not be reconstructed reliably. Consequently, individual feedback exposure could not be quantified or used as a participant-level manipulation check. Future iterations should combine individualized or meal-sensitive threshold calibration with systematic logging of sensor data and feedback events.

Third, both AGI and SGI were active intervention conditions, and no habitual no-guidance baseline was included. The study therefore identifies differences between the two sessions but cannot determine the magnitude of change relative to participants’ natural eating behavior. Future studies should include a regular-tableware condition without behavioral guidance.

Fourth, Groups A, B, and C differed descriptively in age and study setting. Given the small subgroup sizes and the alignment of each group with a particular study setting, age- and setting-related heterogeneity could not be reliably estimated and was not formally tested. Although the within-subject design reduces confounding by stable participant characteristics, the pooled analyses cannot establish that the observed session-level differences were consistent across groups, ages, or study settings. In addition, the study was conducted primarily in campus-related environments and did not cover more diverse contexts such as households or commercial offices. Cultural and environmental factors may influence system acceptance, use, and perceived experience (71). Future studies should therefore evaluate SensEat across a wider range of real-world settings.

Fifth, the sample was relatively small and consisted mainly of young adults familiar with digital technologies, limiting generalizability to other age groups, populations, and cultural settings. The sensitivity analysis indicated a minimum detectable paired effect of *d_z_* = 0.66 under the specified assumptions of *n* = 20, two-sided *α* = 0.05, and 80% power. Effects smaller than this reference value may therefore not have been reliably detected. Accordingly, the nonsignificant differences in Performance, Frustration, Perceived Competence, Effort/Importance, and Pressure/Tension should not be interpreted as evidence of equivalence or the absence of a difference.

Moreover, only three selected NASA-TLX dimensions were analyzed, and no overall NASA-TLX score or predefined acceptability threshold was used. The findings therefore describe differences in Mental Demand, Performance, and Frustration but do not establish that the overall workload was low or acceptable.

Furthermore, the five self-reported experience constructs were each assessed using one study-specific item informed by the IMI, and the three selected NASA-TLX dimensions were analyzed as separate single-item ratings. Internal consistency was therefore not applicable to these single-item measures, and the IMI-informed ratings provide more limited construct coverage than validated multi-item subscales. The corresponding self-reported findings should therefore be interpreted as exploratory. Larger and more diverse samples are needed to provide more precise estimates and detect smaller within-participant differences.

Finally, the study examined only short-term eating behavior and cannot determine whether SensEat supports sustained dietary habit formation. Longer-term field deployments should evaluate continued engagement and system use under adaptive feedback and varied content (72). Because the current system also lacks post-meal reflection and dietary-tracking functions, future versions could provide visual summaries of meal duration and eating pace to support longer-term self-reflection.

## 5 Conclusion

This study presented SensEat, a multimodal embodied tableware system designed to encourage mindful eating during solo dining through implicit and minimally intrusive feedback. By integrating visual, auditory, and haptic cues into everyday tableware, SensEat aims to encourage users’ attention to eating pace within natural dining interactions. Results from the fixed-sequence within-participant comparison indicated that participants had longer meal duration and more chews per bite in the SGI session than in the AGI session, with both behavioral differences remaining statistically significant after Holm correction. These behavioral differences were consistent with a slower eating pace during the SGI session but do not directly demonstrate increased mindful awareness or establish a causal effect of embodied interaction.

Participants also reported significantly higher single-item ratings of Interest/Enjoyment and Value/Usefulness in the SGI session after Holm correction. Interview findings further indicated that participants experienced the embodied interaction as companion-like, supportive, and emotionally comforting during solo dining. For the selected NASA-TLX ratings, Mental Demand was significantly higher in the SGI session, whereas no statistically significant session differences were found for Performance or Frustration after Holm correction. Because no overall workload score or predefined acceptability threshold was used, these results should not be interpreted as demonstrating that the overall cognitive workload was low or acceptable.

This study contributes to Human–Food Interaction at three levels: a modular artifact-embedded feedback architecture; an exploratory, system-level active-comparator account of short-term behavioral, motivational, and workload differences; and design knowledge concerning the trade-off between experiential value and mental demand. The findings further suggest that personalization of cue modality, intensity, and timing is likely to be central to the development of future multimodal tableware systems. However, mindful awareness, self-regulation, long-term dietary habits, and health outcomes were not directly established by the present study. Moreover, because the AGI session always preceded the SGI session, the observed differences may include order, learning, novelty, or repeated-exposure effects. The findings should therefore be interpreted as preliminary evidence of feasibility and as exploratory session-level associations rather than definitive evidence of intervention efficacy. Future studies employing randomized counterbalanced designs, no-guidance baseline conditions, larger and more diverse participant samples, and longer-term field deployments are needed to examine the causal effects, sustained use, and real-world applicability of this approach.

## Data Availability

The original contributions presented in the study are included in the article/Supplementary material, further inquiries can be directed to the corresponding author.
