# Supplementary material for: A multimodal tableware system for supporting mindful eating in solo dining: a pilot study
Source: Front Public Health. 2026 Aug 17;14:1830317. doi: 10.3389/fpubh.2026.1830317 (PMC13527031; doi:10.3389/fpubh.2026.1830317)
Supplement: Supplementary file 1 [file Supplementary_file_1.docx]

Supplementary Material

A Multimodal Tableware System for Supporting Mindful Eating in Solo Dining

**Interview Results**

**RQ1: Participants’ Perceptions of Embodied Multimodal Feedback for Eating-Pace Regulation**

Overall, the majority of participants (16 out of 20) reported preferring the SensEat-guided session to the app-guided session, emphasizing the tangible and immersive qualities of the embodied interaction. Participants described the system as “real, interactive, and enjoyable” (P3) and appreciated its screen-free design: “It feels great not to be guided by a screen while eating” (P11). Several participants also expressed an interest in incorporating this type of interaction into their future eating routines. As P6 noted, “I would be very happy if this system could be integrated into my daily eating routine.” These statements reflect participants’ anticipated willingness to use the system rather than evidence of sustained adoption or long-term behavioral change. Participants frequently described the direct and embodied feedback as helping them notice their eating pace and remain focused on the meal. For example, P2 remarked, “When eating alone, I often scroll on my phone without realizing how fast I eat. SensEat reminded me of staying focused, exactly what I needed.” Similarly, P14 stated, “The multi-layered feedback was very effective for me and helped me stay attentive.” These accounts suggest that participants perceived the multimodal cues as supportive of eating-pace awareness. They were also consistent with the session-level quantitative findings of longer meal duration and more chews per bite during the SGI session than during the preceding AGI session. However, attention and mindful awareness were not directly measured, and the fixed AGI→SGI sequence prevents these differences from being attributed solely to SensEat. Participants also emphasized that the tangible design of the tableware made the guidance feel natural and relatively unobtrusive. Rather than requiring continuous attention to an external screen, SensEat embedded feedback into the dining environment. As P9 explained, “Interacting with the tableware stopped me from staring at the screen and helped me focus on the food itself.” P12 described it as creating a subtle “micro-environment for healthy eating,” adding, “When I follow the rhythm of the tableware, it feels like normal eating. No one would notice that I’m actually being guided.” Taken together, these qualitative accounts indicate that participants generally perceived SensEat’s embodied multimodal feedback as a natural and supportive form of eating-pace guidance. This interpretation is consistent with the longer meal duration and greater number of chews per bite observed during the SGI session (Figure 8). Nevertheless, the present short-term study did not establish increased mindful awareness, sustained behavioral regulation, or long-term habit formation.

**RQ2: Participants’ Perceptions of Enjoyment and Experiential Qualities During SensEat Use**

Participants generally described the SensEat-guided session as more enjoyable and engaging than the preceding app-guided session. The multisensory feedback was frequently characterized as “pleasurable,” “immersive,” and “motivating.” As P11 remarked, “This system feels like a companion sharing the meal with me, rather than just a tool giving instructions.” These qualitative perceptions were consistent with the IMI-informed single-item ratings (Figure 9), in which Interest/Enjoyment and Value/Usefulness were higher during the SGI session than during the AGI session after Holm correction. Because the session order was fixed, however, these differences cannot be attributed solely to the intervention condition. Two recurring experiential qualities were identified in participants’ accounts. First, participants described the multimodal feedback as making eating feel more interactive and embodied. They appreciated the subtlety and rhythm of the cues and perceived them as adding meaning to the eating experience. As P3 commented, “The feedback felt subtle and rhythmic. It made the act of eating more meaningful.” Second, some participants associated SensEat with feelings of achievement and self-reflection. P7 noted, “When my stomach feels comfortable after slow eating, I feel a sense of accomplishment that makes me want to do it again.” Similarly, P16 emphasized, “It helped me experience the benefits of slow eating, which is essential for developing my own eating rhythm.” These comments suggest that some participants perceived the experience as motivational. They should not, however, be interpreted as evidence that SensEat produced sustained intrinsic motivation or long-term behavioral adherence. Participants also described SensEat as socially friendly and emotionally supportive during solo dining. For example, P6 stated, “It helped me stay focused while eating alone. It felt like something was sharing the moment with me.” Such descriptions suggest that the embodied cues sometimes evoked a short-term sense of companionship or shared presence. These accounts represent participants’ subjective interpretations of the interaction rather than evidence that SensEat created an actual social relationship, reduced loneliness, or improved emotional well-being. Overall, the qualitative findings were consistent with the higher Interest/Enjoyment and Value/Usefulness ratings observed during the SGI session. They further illustrate how participants interpreted the embodied feedback as enjoyable, meaningful, and occasionally companion-like. However, these short-term experiential accounts do not establish increased mindful awareness, sustained motivation, or long-term changes in eating behavior.

**RQ3: Participants’ Perceptions of Workload and Attentional Demand During SensEat Use**

The selected NASA-TLX results showed that Mental Demand was significantly higher during the SGI session than during the AGI session, whereas no statistically significant session differences were found for Performance or Frustration after Holm correction (Figure 10). The qualitative findings provided additional context for understanding how participants experienced this increased attentional demand during SensEat use. Many participants described the sensory prompts as “gentle” and “non-intrusive,” although some acknowledged that SensEat required greater attention to the eating process. P4 explained, “It made me more focused, but not in a stressful way—it felt like a natural rhythm.” Similarly, P10 remarked, “The feedback reminded me of slowing down, but it was soft and didn’t interrupt my meal.” P12 also described a perceived balance between awareness and relaxation: “I could notice my eating pace, but I didn’t feel tense or overloaded.” At the same time, participants did not experience the feedback uniformly. Some reported occasional distraction when several cues, including light, vibration, and sound, occurred simultaneously. P8 stated, “At first, I paid too much attention to the signals, but after a while it felt natural. I didn’t have to think about it anymore.” This account suggests that the interaction became more familiar to this participant during the experimental session, although the short study duration does not establish longer-term adaptation or reduced cognitive demand over time. Taken together, the qualitative findings suggest that many participants perceived the additional attentional demand as manageable during the short SGI session, while some experienced temporary distraction or overstimulation. These accounts should be interpreted alongside the significantly higher Mental Demand score observed during SGI. Because only three selected NASA-TLX ratings were analyzed separately and no overall workload score or predefined acceptability threshold was used, the present findings do not establish that SensEat imposed a low or acceptable overall cognitive workload, nor do they demonstrate the absence of cognitive overload.

**Figure Legends**

**Figure S1. Individual paired behavioral outcomes in the AGI and SGI sessions.** (a) Chews per bite; (b) meal duration. Each line connects the AGI and SGI observations from the same participant (n = 20). Diamonds show mean ± SD for chews per bite and median [IQR] for meal duration. Holm-adjusted p values are shown.

**Figure S2. Individual paired observations for the five IMI-informed single-item ratings in the AGI and SGI sessions.** (a) Interest/Enjoyment; (b) Value/Usefulness; (c) Perceived Competence; (d) Effort/Importance; and (e) Pressure/Tension. Each line connects the AGI and SGI observations from the same participant (n = 20). Diamonds show median [IQR] for Interest/Enjoyment, Value/Usefulness, and Effort/Importance, and mean ± SD for Perceived Competence and Pressure/Tension. Holm-adjusted p values are shown.

**Figure S3. Individual paired observations for the three selected NASA-TLX ratings in the AGI and SGI sessions.** (a) Mental Demand; (b) Performance; and (c) Frustration. Each line connects the AGI and SGI observations from the same participant (n = 20). Diamonds show median [IQR] for Mental Demand and mean ± SD for Performance and Frustration. Holm-adjusted p values are shown. For the adapted Performance scale used in this study, higher scores indicate better perceived performance. For Mental Demand and Frustration, higher scores indicate greater perceived demand or frustration.

Figure S1

Figure S2

Figure S3
